# Comparisons of recurrence-free survival and overall survival between microwave versus radiofrequency ablation treatment for hepatocellular carcinoma: A multiple centers retrospective cohort study with propensity score matching

**DOI:** 10.1371/journal.pone.0227242

**Published:** 2020-01-09

**Authors:** Shibin Du, Jian-Zhi Yang, Jing Chen, Wei-gang Zhou, Yan-Yan Sun

**Affiliations:** 1 Department of Anesthesiology, Shenzhen University Clinical Medical Academy, Shenzhen University, Shenzhen University General Hospital, Shenzhen, China; 2 Department of Anesthesiology, Hanzhong Central Hospital, Hanzhong, Shanxi, China; 3 Department of Endocrinology, The Second Affiliated Hospital, Kunming Medical University, Kunming, Yunnan, China; University of Cincinnati College of Medicine, UNITED STATES

## Abstract

Both microwave (MW) ablation and radiofrequency (RF) ablation are widely used for hepatocellular carcinoma (HCC) treatments in clinic. However, it is still unclear if ablative methods could influence the recurrence-free survival (RFS) and overall survival (OS) of HCC patients. Therefore, we carried out this multi-center retrospective cohort study to investigate the differences of recurrence-free survival (RFS) and overall survival (OS) between MW ablation and RF ablation by survival analysis. From January 2014 to December 2016, patients who received thermal ablation surgery for HCC treatment were screened. Finally, 452 patients met the eligibility criteria and finished the follow-up. Univariable and multivariable regression analyses were used to identify independent predictive factors of the RFS and OS. Also, propensity score matching (PSM) was used to balance the bias between two groups. Finally, we found that before the PSM, the univariable and multivariable regression analyses revealed that there were no significant differences on the RFS between two groups. Same results were obtained for the OS. After PSM, 115 pairs of patients were created, and both the univariable and multivariable regression analyses suggested that there were still no significant differences on the RFS between two groups. Same results were obtained for the OS. In conclusion, our present study showed that there were no significant differences between MW ablation and RF ablation for HCC patients on the RFS or OS.

## Introduction and background

Hepatocellular carcinoma (HCC) is the fifth most common malignant tumor and the third leading cause of cancer-related death worldwide [1,2]. Especially in Southeast Asia and Africa, the incidence rate and mortality rate of HCC are significantly higher than other areas [3]. In China, HCC is the third incidence rate and mortality rate for all cancers [4]. Though, in recent decades, it showed appreciable declines in rates of HCC in China, the mortality was still two- to five-fold higher than in most European countries and the Americas [5,6]. Related statistic data showed that China accounted for more than 50% of the deaths from liver cancer worldwide [7]. So, HCC and HCC treatments are still pressing problems in China.

Till now, diverse treatments have been applicated in overcoming HCC [8,9]. For example, hepatic resection, microwave ablation, radiofrequency ablation, biotherapy and transcatheter arterial chemoembolization. Surgical resection is currently the primary treatment for HCC. However, with the development of percutaneous ablation surgery, microwave (MW) ablation and radiofrequency (RF) ablation for curing HCC have been widely used in clinic. Compared with hepatic resection, MW ablation and RF ablation have some advantages such as speediness, lower injury, fast in recovery [10]. More important, MW ablation and RF ablation are more suitable for patients who had severe liver cirrhosis or locations of tumors were close to vessels.

Numerous studies have compared the efficacy and safety between MW ablation and RF ablation [11–15]. Some studies suggested that MW ablation was better than RF ablation and it seemed to lead a better prognosis for MW ablation. For example, a prospective study involved 111 patients who received MW ablation or RF ablation suggested that a lower incidence of local recurrence was observed in microwave group [11]. However, other studies revealed that there was no difference between MW ablation and RF ablation. A meta-analysis which involved 2062 patients showed that MW ablation and RF ablation had similar 1–5-year overall survival, disease-free survival, local recurrence rate, and adverse events [12]. It is currently unclear and lack of rigorous proof to recommend one ablative method. Therefore, we carried out this multi-center retrospective cohort study to investigate the differences of recurrence-free survival (RFS) and overall survival (OS) between MW ablation and RF ablation by survival analysis. We hypothesize that there are no significant differences between MW ablation and RF ablation for HCC patients’ prognosis.

## Patients and methods

### Patients selection

**Fig 1:**
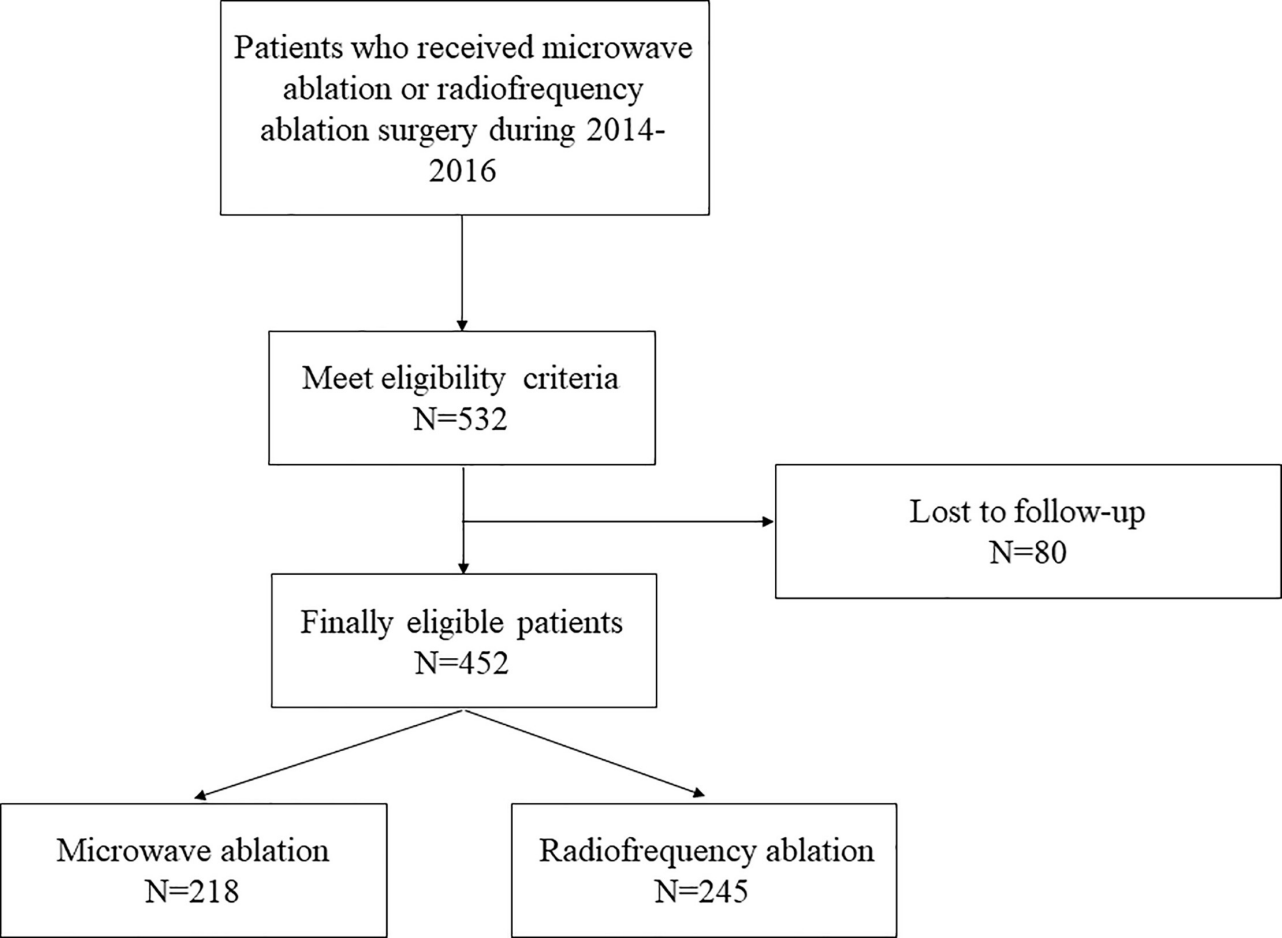
Flow chart showing how the cohort including 532 patients was generated for analysis in this study.

Patients who underwent MW ablation or RF ablation for HCC in Shenzhen University General Hospital, Hanzhong Central Hospital and The Second Affiliated Hospital of Kunming Medical University from January 2014 to December 2016 were screened. Finally, 532 patients accorded with the eligibility criteria and were enrolled into the study (**Fig 1**). This study was conducted in accordance with the Declaration of Helsinki and was approved by the Clinical Research Ethics Committee of the Shenzhen University General Hospital, and received approval from other two hospitals (the Ethics Committee of Hanzhong Central Hospital and the Ethics Committee of The Second Affiliated Hospital of Kunming Medical University). Also, a waiver of written consent was approved by the Clinical Research Ethics Committee of the Shenzhen University General Hospital.

### Inclusion and exclusion criteria

Eligible patients who met the inclusion and exclusion criteria would be enrolled, and related data would be collected by trained researchers (**Table 1**). The diagnoses of HCC were confirmed by postoperative histopathology. Severe organ failure mainly includes liver failure (Child-Pugh C degree), heart failure (NYHA Ⅲ-Ⅳ), and renal failure (serum creatinine>442 μmol/L). Severe immune system disease mainly includes immunodeficiency disease, systemic lupus erythematosus, rheumatoid arthritis and ankylosing spondylitis.

**Table 1:** Study eligibility criteria.

Inclusion criteria • Age: 18–75 years • Race: all racial groups • American Society of Anesthesiologists degree: Ⅰ-Ⅲ • Primary liver cancer • Child-Pugh degree: A or B • No macrovascular vessels were invaded by tumor • No distal lymph node or extrahepatic metastasis • Patients who received microwave ablation or radiofrequency ablation surgery as the first choice after liver cancer was found Exclusion criteria • A history of liver surgery • A combined surgery of liver resection surgery and microwave ablation / radiofrequency ablation surgery • Had severe organ failure or immune system disease • The sum of the long diameter of individual hepatocellular carcinoma or multiple hepatocellular carcinoma> 5 cm • Metastatic liver cancer • Had severe postoperative complications: massive haemorrhage (>200 ml), infection, diaphragmatic injury and biliary tract injury. • A history of any kinds of cancers or currently associated with any kinds of cancers

### Follow-up

All patients were reexamined using serum alpha fetoprotein (AFP), ultrasound or CT, and chest X-ray at 1 month after surgery. Then, patients were followed-up at a 2-monthly interval for the first 6 months and at a 3-monthly interval thereafter. Tumor recurrence was defined as new appearance of intra- or extrahepatic tumor nodule. The clinical practice guidelines of EASL-EORTC was used for the diagnosis of tumor recurrence [16]. Patients with tumor recurrence were actively treated with percutaneous ablative, hepatic resection, transcatheter arterial chemoembolization (TACE), radiotherapy or conservative treatment.

The follow-up began from January, 2014 and ended at March, 2018. All data used for analysis was collected from digital medical history or paper medical records, and all data were fully anonymized before access. In every center, two trained researchers were in charge of follow-up and all data were entered using “Epidata”.

### Variables and outcomes

In this study, 22 variables were collected and analyzed. All variables could be divided as patient characteristics (age, sex, ASA score, hypertension, diabetes and cardiopathy), liver function variables (cirrhosis, HBV/HCV infection, child-pugh stage, AFP, TBiL, ALB, ALT, and AST), operative variables (tumor number, tumor size, anesthetic methods and adjuvant chemoradiotherapy) and follow-up information (dates of operation, inpatient days, dates of tumor recurrence and dates of death). All data were collected and entered with the same way as the follow-up data. In every center, two trained researchers were in charge of collecting data.

The main outcome of this study was RFS and the second outcome was OS. The RFS was defined from the date of the surgery to the date of first recurrence. If the recurrence of tumor was not recorded, the RFS was defined as the time between the date of surgery and the date of last follow-up. The OS was calculated from the date of the surgery to either the date of death or the date of the last follow-up visit.

### Propensity score matching

Because patients were not randomly allocated to the MW ablation group and RF ablation group, and variables in two groups were imbalanced. We decided to use the propensity score matching as described before [17] to eliminate the imbalance between two groups. This method consisted of ordering the case and control subjects, then selecting the first case subject and finding the control subject with the closest propensity score [18]. A logistic regression model was built given the covariates of tumor number, cirrhosis, HBV/HCV infection, Child-Pugh stage, AFP, ALB, TBIL, anesthetic methods, tumor size, adjuvant chemoradiotherapy and hypertension, and the dependent variable of ablation methods. We applied 1:1 nearest neighbor matching without replacement to ensure that conditional bias was minimized. For each patient having MW ablation, a patient having RF ablation with a minimum in distance of propensity scores was matched. The caliper width was 0.05 for propensity score matching. Propensity score matching was carried out using IBM SPSS Statistics 23.0 version.

### Statistical analysis

Statistical analyses were carried out using the IBM SPSS Statistics 23.0 (SPSS Inc., Armonk, NY, USA). Categorical variables were reported as number (n) or proportion (%) and continuous variables were expressed as mean ± standard deviation (SD) or median (range). The Student’s t test was used for comparisons of continuous variables. Otherwise, the Mann-Whitney U test was applied. Categorical variables were compared with the χ^2^ test with the Yates correction or the Fisher’s exact test, as appropriate. To identify independent predictive factors of prognosis, univariable and multivariable regression analyses were used. The RFS and OS rates were compared between the MW ablation and RF ablation groups before and after propensity matching using the Kaplan-Meier regression analyses or univariable Cox regression analyses. Multivariable Cox proportional hazard regression analyses were then performed to adjust for other prognostic factors which were associated with OS and RFS [19]. All statistical tests were 2 sided, and *P* values <0.05 were considered statistically significant.

## Result

532 patients who underwent MW or RF ablation met the eligibility criteria, and finally 452 patients finished follow-up. Patients were divided into two groups: the MW ablation group (N = 218, 48.2%), and the RF ablation group (N = 234, 51.8%). The comparisons of patients’ characteristics and other variables between two groups in the entire cohort are illustrated in **Table 2**. Patients’ characteristics including Hypertension, Tumor size and Anesthetic methods are significantly different between two groups (*P*<0.05). The follow-up time was at a range of 1.25- to 4.25-year, and the average follow-up time was 2.34-year.

**Table 2:** Comparisons of patients’ characteristics and other variables between MW ablation group and RF ablation group in the entire cohort.

Variables	Before PSM*			*After PSM*		
	MW Group	RF Group	*P* value	MW Group	RF Group	*P* value
PS score	0.29 (0.22)	0.43 (0.21)	0.000	0.46 (0.21)	0.48 (0.21)	0.641
Sex						
male	173 (79.4%)	192 (82.1%)	0.468	92 (80.0%)	88 (76.5%)	0.525
female	45 (20.6%)	42 (17.9%)		23 (20.0%)	27 (23.5%)	
Age	56.4±10.3	57.3±9.3	0.323	56.3±10.0	57.5±9.4	0.315
≤60	137 (62.8%)	143 (61.1%)	0.705	74 (64.3%)	69 (60.0%)	0.499
>60	81 (37.2%)	91 (38.9%)		41 (35.7%)	46 (40.0%)	
ASA score*						
Ⅱ	169 (77.5%)	195 (83.3%)	0.119	88 (76.5%)	87 (75.7%)	0.878
Ⅲ	49 (22.5%)	39 (16.7%)		27 (23.5%)	28 (24.3%)	
Hypertension (Yes/No)	41/177	68/166	0.011	26/89	37/78	0.105
Diabetes (Yes/No)	31/187	31/203	0.198	17/98	19/96	0.718
Cardiopathy** (Yes/No)	5/213	14/220	0.051	2/113	7/108	0.171
Tumor number						
1	177 (81.2%)	202 (86.3%)	0.333	96 (83.5%)	95 (82.6%)	0.983
2	37 (17.0%)	29 (12.4%)		17 (14.8%)	18 (15.7%)	
3	4 (1.8%)	3 (1.3%)		2 (1.7%)	2 (1.7)	
Cirrhosis (Yes/No)	176/42	185/49	0.657	85/30	87/28	0.763
HBV/HCV* infection (Yes/No)	190/28	213/32	0.945	98/17	97/18	0.855
Adjuvant chemoradiotherapy***						
(Yes/No)	28/190	90/144	0.000	19/96	26/89	0.247
Child-Pugh stage						
A	200 (91.7%)	216 (92.3%)	0.825	107 (93.0%)	105 (91.3%)	0.625
B	18 (8.3%)	18 (7.7%)		8 (7.0%)	10 (8.7%)	
Tumor size	2.9±1.2	2.4±1.0	0.000	2.4±1.1	2.6±1.1	0.465
<3cm	110 (50.5%)	166 (70.9%)	0.000	80 (69.6%)	74 (64.3%)	0.400
≥3cm	108 (49.5%)	68 (29.1%)		35 (30.4%)	41 (35.7%)	
Anesthetic methods						
General anesthesia	56 (25.7%)	155 (66.2%)	0.000	31 (27.0%)	40 (34.8%)	0.201
Local anesthesia	162 (74.3%)	79 (33.8%)		84 (73.0%)	75 (65.2%)	
AFP*						
<400ng/ml	179 (82.1%)	201 (85.9%)	0.272	101 (87.8%)	98 (85.2%)	0.564
≥400ng/ml	39 (17.9%)	33 (14.1%)		14 (12.2%)	17 (14.8%)	
TBIL*						
<34mmol/L	201 (92.2%)	211 (90.2%)	0.447	106 (92.2%)	103 (89.6%)	0.494
≥34mmol/L	17 (7.8%)	23 (9.8%)		9 (7.8%)	12 (10.4%)	
ALB*						
≤35g/L	32 (14.7%)	36 (15.4%)	0.834	11 (9.6%)	14 (12.2%)	0.527
>35g/L	186 (85.3%)	198 (84.6%)		104 (90.4%)	101 (87.8%)	
ALT*						
<40U/L	128 (58.7%)	144 (61.5%)	0.540	72 (62.6%)	63 (54.8%)	0.230
≥40U/L	90 (41.3%)	90 (38.5%)		43 (37.4%)	52 (45.2%)	
AST*						
<40U/L	134 (61.5%)	133 (56.8%)	0.317	75 (65.2%)	65 (50.4%)	0.177
≥40U/L	84 (38.5%)	101 (43.2%)		40 (34.8%)	50 (49.6%)	
Inpatient days^#^						
≤5 days	213 (97.7%)	222 (94.9%)	0.113	113 (98.3%)	107 (93.0%)	0.102
>5 days	5 (2.3%)	12 (5.1%)		2 (1.7%)	8 (7.0%)	

*ASA, American Society of Anesthesiologists; HBV, hepatitis B virus; HCV, hepatitis C virus; AFP, alpha-fetoprotein; TBIL, total bilirubin; ALB, serum albumin; ALT, Alanine transaminase; AST, aspartate aminotransferase; PSM, propensity score matching.

** Cardiopathy illnesses include coronary heart disease, heart failure (NYHA Ⅰ-Ⅱ), arrhythmia, myocardiopathy and valvulopathy.

*** Adjuvant chemoradiotherapy includes transcatheter arterial chemoembolization (TACE) and radioactive seed implantation. Adjuvant chemoradiotherapy is defined as patients received TACE or radioactive seed implantation during the hospital admission for first treatment.

^#^Inpatient days is defined as the period from operation finished to hospital discharge.

In our retrospective study, all patients received ablation surgery by percutaneous approach. Data regarding the complete response, differentiation between local and distant recurrence, need for repeat ablations, postoperative complications and Edmandson grade were showed in **Table 3**. No statistical differences were observed between two groups.

**Table 3:** Data of the complete response, recurrence, repeat ablations, postoperative complications and Edmandson grade.

	MW Group	RF Group	*P* value
Complete response			
Yes	193 (88.5%)	200 (85.5%)	0.334
No	25 (11.5%)	34 (14.5%)	
Recurrence			
Local	106 (89.8%)	120 (89.5%)	0.942
Distant	12 (10.2%)	14 (10.5%)	
Repeat ablations (Yes/No)	91/27	111/23	0.256
Postoperative complications			0.618
Fever	7	10	
Seroperitoneum	1	2	
Pain	13	13	
Skin burn	3	1	
Edmandson grade			0.214
Ⅰ	28	32	
Ⅱ	122	147	
Ⅲ	65	50	
Ⅳ	3	5	

**Fig 2:**
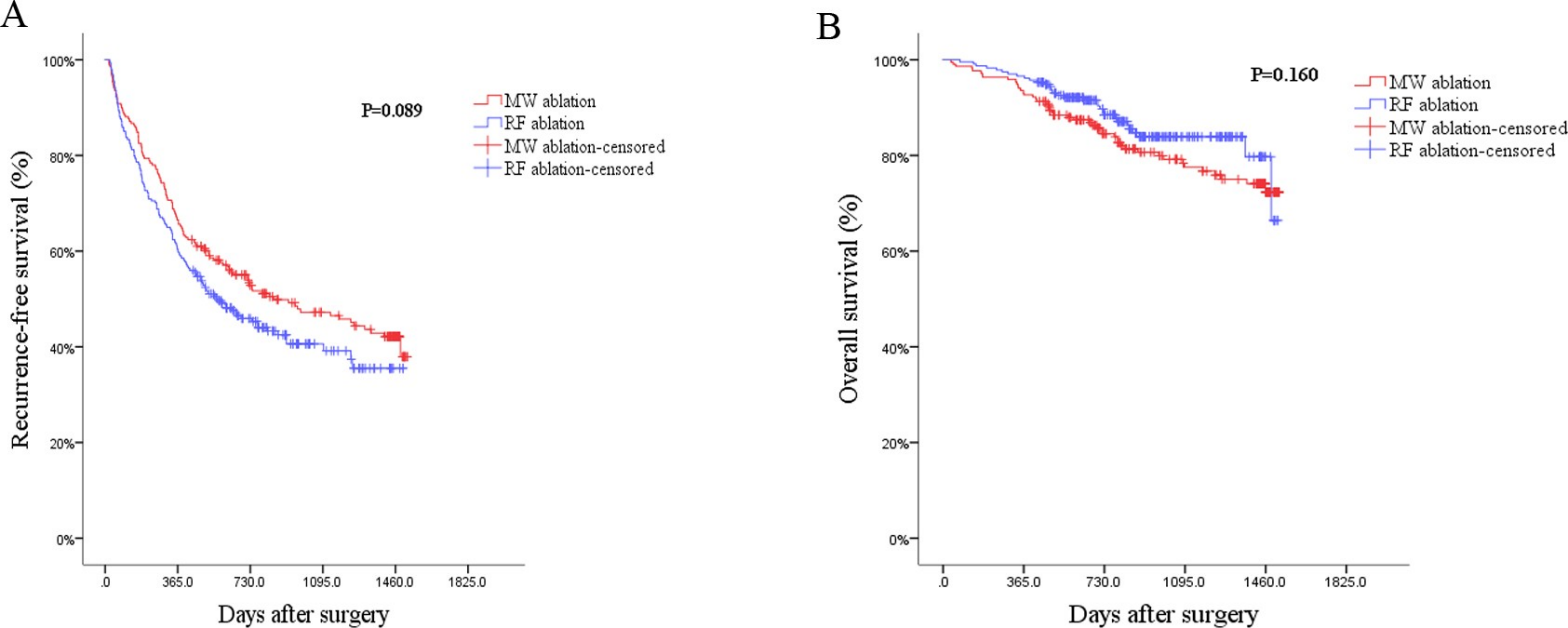
2A: Log-rank test showed on difference between the two groups in recurrence-free survival; 2B: Log-rank test showed on difference between the two groups in overall survival.

First, we used Kaplan-Meier analysis or univariable Cox regression model analysis to screen variables which had significant influence on the RFS and OS. From the **Table 4** showed that ASA score, Hypertension, Tumor number, Cirrhosis, Adjuvant chemoradiotherapy, AFP, Tumor size and anesthetic methods would significantly influence patients’ RFS (*P*<0.05). However, we found that the Ablation methods had no observable influence on the RFS by log-rank test (**Fig 2A,** *P* = 0.089). Also, results suggested that Age, ALB, Child-Pugh stage, Tumor size and anesthetic methods could remarkably influence the OS. But the ablation methods had no significant influence on the OS by log-rank test, too (**Fig 2B,** *P* = 0.160).

**Table 4:** Results of univariable Cox regression model analysis on RFS and OS.

Univariable Cox regression	HR (95% CI)*	*P* value
RFS*		
ASA score*	0.619 (0.463–0.826)	0.001
Hypertension	0.704 (0.534–0.927)	0.013
Tumor number	0.504 (0.389–0.652)	0.000
Cirrhosis	0.680 (0.486–0.950)	0.024
Adjuvant chemoradiotherapy	0.563 (0.430–0.736)	0.000
AFP*	0.716 (0.519–0.989)	0.043
Tumor size	0.532 (0.414–0.682)	0.000
Anesthesia methods	0.640 (0.498–0.822)	0.000
Ablation methods	1.241 (0.967–1.593)	0.089
OS*		
Age	0.617 (0.396–0.959)	0.032
ALB*	0.465 (0.282–0.766)	0.003
Child-Pugh stage	0.449 (0.237–0.850)	0.014
Tumor size	0.445 (0.285–0.693)	0.000
Anesthesia methods	0.632 (0.402–0.993)	0.046
Ablation methods	0.723 (0.459–1.139)	0.160

*RFS, recurrence-free survival; OS, overall survival; HR, hazard ratio; CI, confidence interval; ASA, American Society of Anesthesiologists; AFP, alpha-fetoprotein; ALB, serum albumin.

Significant variables (*P*<0.05) as shown in **Table 4** were entered into multivariable Cox regression model analysis. As the **Table 5** showed, the ablation methods had no significant influence on the RFS and OS (both *P*>0.05). It suggested that different ablation methods would not influence the RFS or OS for HCC patients.

**Table 5:** Multivariable Cox regression model analysis of RFS and OS.

Independent predictive factor	HR (95% CI)	*P* value
**RFS**		
ASA score	0.722 (0.531–0.982)	0.038
Tumor size	0.653 (0.491–0.870)	0.004
Adjuvant chemoradiotherapy	0.694 (0.514–0.937)	0.017
Hypertension	0.743 (0.555–0.996)	0.047
AFP	0.720 (0.519–1.000)	0.050
Tumor number	0.611 (0.464–0.809)	0.000
Ablation methods	0.872 (0.638–1.192)	0.390
**OS**		
Age	0.603 (0.386–0.943)	0.026
Tumor size	0.526 (0.328–0.841)	0.007
Ablation methods	0.672 (0.400–1.129)	0.133

**Fig 3:**
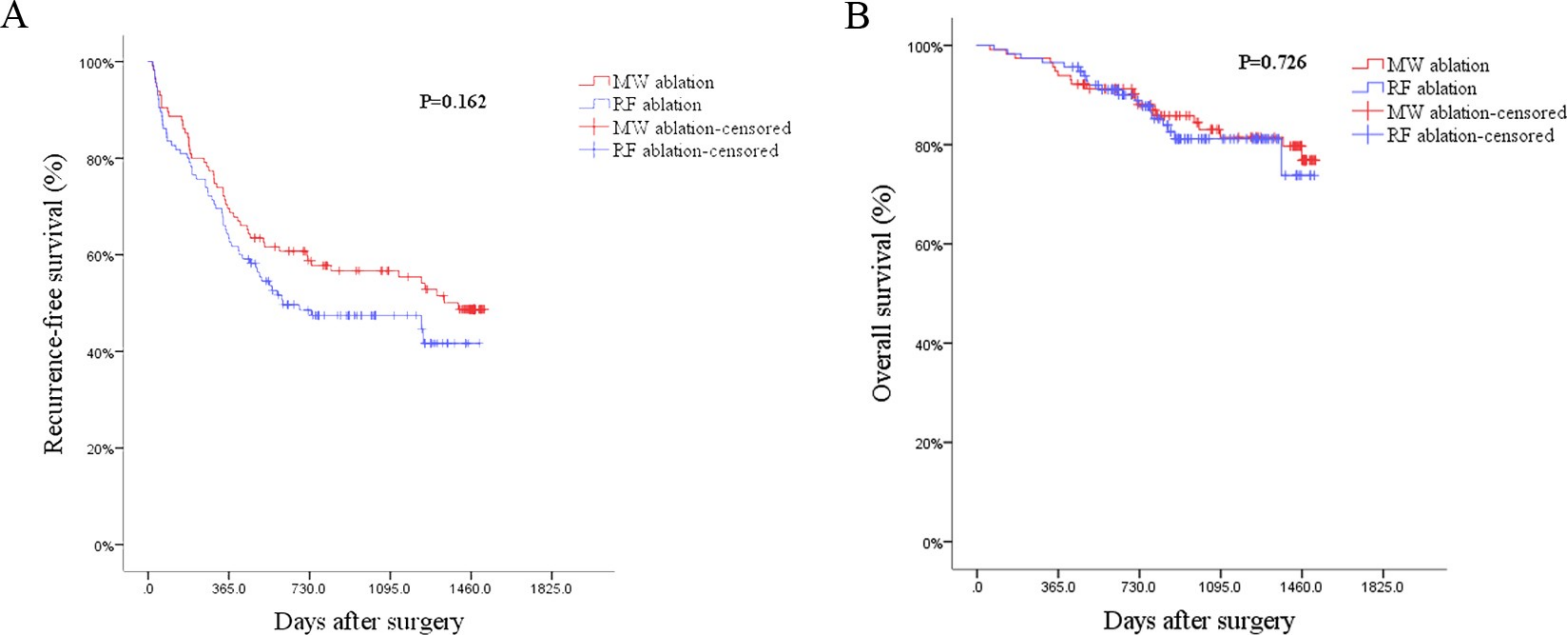
3A: Log-rank test showed on difference between the two groups in recurrence-free survival after PSM. 3B: Log-rank test showed on difference between the two groups in overall survival after PSM.

PSM analysis was carried out as illustrated above and finally created 115 pairs of patients. After the PSM, there no significant differences of variables and PS score between two groups (**Table 2**). Comparisons of patients’ RFS and OS between two groups after PSM were shown in **Table 6**. It suggested again that different ablation methods would not influence the RFS (**Fig 3A,** *P* = 0.162) or OS of HCC patients (**Fig 3B,** *P* = 0.726). Results in multivariable Cox regression model analysis showed that there were no differences on the RFS and OS after PSM (**Table 7**).

**Table 6:** Results of univariable Cox regression model analysis on RFS and OS after PSM.

Univariable Cox regression	HR (95%CI)	*P* value
**RFS**		
ASA	0.578 (0.390–0.857)	0.006
Hypertension	0.651 (0.441–0.960)	0.030
Diabetes	0.518 (0.332–0.808)	0.004
Tumor number	0.403 (0.265–0.612)	0.000
Cirrhosis	0.591 (0.368–0.948)	0.029
Adjuvant chemoradiotherapy	0.641 (0.415–0.989)	0.045
AFP	0.520 (0.324–0.835)	0.007
Tumor size	0.446 (0.307–0.649)	0.000
Anesthesia method	0.617 (0.420–0.907)	0.014
Ablation method	0.769 (0.532–1.111)	0.162
**OS**		
Age	0.407 (0.216–0.768)	0.006
Tumor size	0.360 (0.189–0.687)	0.002
Anesthesia method	0.576 (0.413–0.804)	0.001
Inpatient day	0.347 (0.123–0.983)	0.046
Ablation method	0.892 (0.470–1.691)	0.726

**Table 7:** Multivariable Cox regression model analysis of RFS and OS after PSM.

Multivariable Cox regression	HR (95%CI)	*P* value
**RFS**		
Tumor number	0.521 (0.330–0.823)	0.005
AFP	0.496 (0.307–0.802)	0.004
Tumor size	0.565 (0.338–0.945)	0.030
Ablation method	0.890 (0.603–1.313)	0.558
**OS**		
Age	0.390 (0.205–0.741)	0.004
Anesthesia method	0.422 (0.195–0.912)	0.028
Ablation method	1.169 (0.596–2.291)	0.65l

Before the PSM, the Kaplan–Meier survival rate estimates and 95% CIs at landmark follow-up times showed that the 1-, 2-, 3- and 4-year RFS rates of tumor in MW ablation group were 66.5% (95%CI, 60.2%-72.8%), 52.8% (95%CI, 46.1%-59.5%), 47.2% (95%CI, 40.3%-54.1%) and 42.1% (95%CI, 34.8%-49.4%) respectively; In RF ablation group, the 1-, 2-, 3- and 4-year RFS rates of tumor were 60.3% (95%CI, 54.0%-66.6%), 45.9% (95%CI, 39.4%-52.4%), 40.6% (95%CI, 33.7%-47.5%) and 35.5% (95%CI, 27.5%-43.5%) respectively (**Table 8**). The log-rank test showed that there was no statistic difference on the RFS rates between two groups (**Fig 2A**). Same conclusion could be affirmed again for the OS rates between MW ablation and RF ablation groups (**Table 8 and Fig 2B**).

**Table 8:** The RFS rates and survival rates of patients before PSM. (*Point-wise 95% CI).

Time	MW ablation group (*n* = 218)				RF ablation group (*n* = 234)			
	RFS rates (95% CI*)	No. events	No. censored	No. left	RFS rates (95% CI*)	No. events	No. censored	No. left
At treatment	100			218	100			234
1 yr	66.5 (60.2–72.8)	73	0	145	60.3 (54.0–66.6)	93	0	141
2 yr	52.8 (46.1–59.5)	101	24	93	45.9 (39.4–52.4)	124	34	76
3 yr	47.2 (40.3–54.1)	110	41	67	40.6 (33.7–47.5)	131	60	43
4 yr	42.1 (34.8–49.4)	117	72	29	35.5 (27.5–43.5)	134	97	3
	OS rates (95% CI*)	No. events	No. censored	No. left	OS rates (95% CI*)	No. events	No. censored	No. left
At treatment	100			218	100			234
1 yr	92.7 (89.2–96.2)	16	0	202	96.6 (94.2–99.0)	8	0	226
2 yr	84.5 (79.6–89.4)	32	47	139	88.5 (84.0–93.0)	24	69	141
3 yr	77.5 (71.2–83.8)	42	81	95	83.9 (78.4–89.4)	30	148	56
4 yr	72.3 (64.9–79.7)	47	132	39	79.7 (70.1–89.3)	31	196	7

## Discussion

According to this multi-center retrospective cohort study, we found that there were no significant differences between MW ablation and RF ablation for HCC patients on the RFS or OS. Though liver resection is the first-line curative treatment for patients with HCC, several studies had verified that hepatic resection contributed to a higher rate of complications and surgical mortality [20,21]. At the same time, more studies also found that ablation surgery was equivalent to surgical resection for overall survival [22,23]. In conclusion, ablation surgery is a kind of effective and less invasive method for tumor treatments.

However, with the development of MW ablation and RF ablation, researchers focus on the differences between two methods. Some studies [11,24,25] reported that MW ablation seemed to have a lower rate of local recurrence of tumor. It could be explained that MW ablation has an improved convection profile, higher intratumoral temperatures, faster ablation time, larger ablation volume, and less susceptibility to heat-sink effect [26,27]. But still other studies found that no significant differences on the RFS or OS were observed between MW ablation group and RF ablation group [28,29]. In our study, we further verified that there were similar RFS and OS for HCC patients in MW ablation group and RF ablation group. In a word, RF ablation and MW ablation have same clinical value in treating HCC conforming to the Milan criteria, and these two methods are both safe and effective techniques for HCC as clinical applications.

In this study, more than 20 related variables were collected and analyzed. After PSM, results of multiple Cox regression analysis showed that AFP, Tumor size and Tumor number were independent risk factors for the RFS, and the Anesthetic methods and Age were independent risk factors for the OS. In this study, we found that the local anesthesia was better than general anesthesia, and the OS in local anesthesia group was longer than the general anesthesia group. This finding could be verified by related studies [30,31].

This study has several limitations. First, it is a retrospective cohort study rather a randomized controlled trail. But we had used the PSM and multiple Cox regression analyses to minimize the bias between two groups. Second, the follow-up time could be longer. In the further study, we would prolong the follow-up period to obtain more information about the RFS and OS. Third, the sample capacity could be larger and more hospitals are needed. Fourth, some variables could be collected with more details. For example, because different hospitals have diverse medical record software, some data was missing during the replacement of software. And some data about the dosage of anesthetic drugs and adjuvant chemotherapeutic drugs were not recorded with details, so we could not collect relevant data intactly.

In summary, after using PSM analyses and multivariable Cox regression analyses, our present study showed that there were no significant differences between MW ablation and RF ablation for HCC patients on the RFS or OS. Both MW ablation and RF ablation were effective and safe for patients who suffered HCC.
